# Molecular evolution of the hemagglutinin and neuraminidase genes of pandemic (H1N1) 2009 influenza viruses in Sendai, Japan, during 2009–2011

**DOI:** 10.1007/s11262-013-0980-5

**Published:** 2013-09-29

**Authors:** Irona Khandaker, Akira Suzuki, Taro Kamigaki, Kentaro Tohma, Takashi Odagiri, Takashi Okada, Ayumu Ohno, Kanako Otani, Rumi Sawayama, Kazuhisa Kawamura, Michiko Okamoto, Hitoshi Oshitani

**Affiliations:** 1 Department of Virology, Tohoku University Graduate School of Medicine, 2-1 Seiryomachi, Aoba, Sendai, Miyagi 9807585 Japan; 2 Kawamura Pediatric Clinic, 2-22-27, Saiwaicho, Miyagino-Ku, Sendai, Miyagi 983-0836 Japan

**Keywords:** A(H1N1)pdm09, Neuraminidase, Oseltamivir-resistant, Antiviral drug susceptibility, Positive selection

## Abstract

Analyzing the evolutionary pattern of the influenza A(H1N1)pdm09 strain in different regions is important for understanding its diversification. We therefore conducted this study to elucidate the genetic variability and molecular evolution of the influenza A(H1N1)pdm09 strains that circulated during the 2009–2010 and 2010–2011 influenza seasons in Sendai, Japan. Nasopharyngeal swab specimens were collected from patients with influenza-like illnesses who visited outpatient clinics in Sendai City, Japan, from September 2009 to April 2011. A total of 75 isolates were selected from September 2009 to April 2011 to analyze the genetic changes in the entire hemagglutinin 1 (HA1) segment of the HA gene and the neuraminidase (NA) gene based on sequence analysis. Bayesian coalescent Markov chain Monte Carlo analyses of HA1 and NA gene sequences were performed for further analysis. High sequence identities were observed for HA1 and NA in influenza A(H1N1)pdm09, displaying 99.06 and 99.33 % nucleotide identities, respectively, with the A(H1N1)pdm09 vaccine strain A/California/07/2009. The substitution rates of nucleotides for HA1 in the 2009–2010 and 2010–2011 were 1.5 × 10^−3^ and 1.6 × 10^−3^ substitutions per site per year, respectively. Phylogenetic tree analysis demonstrated that Sendai isolates were clustered into global clade 7, which is characterized by an S203T mutation in the HA1 gene. Moreover, two distinct circulation clusters were present in the 2010–2011 season. Mutations were present in antigenic or receptor-binding domains of the HA1 segment, including A141V, S143G, S183P, S185T, and S203T. The Bayesian skyline plot model illustrated a steady rate for the maintenance of genetic diversity, followed by a slight increase in the later part of the 2010–2011 season. Selection analysis revealed that the HA1 (position 197) and NA (position 46) sites were under positive selection; however, no known mutation conferring resistance to NA inhibitors such as H275Y was observed. The effect on control of the influenza A(H1N1)pdm09 virus, including vaccine strain selection, requires continuous monitoring of the strain by genetic surveillance.

**Electronic supplementary material:**

The online version of this article (doi:10.1007/s11262-013-0980-5) contains supplementary material, which is available to authorized users.

## Introduction

The first influenza pandemic of this century occurred in April 2009 with the emergence of a novel A(H1N1)pdm09 strain in Mexico and the USA [1]. The virus then spread worldwide, affecting more than 213 countries with 425,650 laboratory-confirmed cases and at least 16,813 deaths by March 2010 [2]. The first community transmission of influenza A(H1N1)pdm09 was confirmed in Japan in May 2009 among high school students without any history of travel to countries with local transmission [3]. By the end of July of the same year, more than 5,000 cases had been reported in Japan [4], and the disease had spread throughout the country. Owing to its overwhelming public health implications, understanding the evolution of the influenza A virus is of great interest for control strategies such as vaccine development.

The influenza A virus is an RNA virus belonging to the family *Orthomyxoviridae*. The virus genome consists of eight gene segments. Among these segments, segments 4 and 6 encode the viral surface glycoproteins hemagglutinin (HA) and neuraminidase (NA), respectively. HA is responsible for binding to sialic acids (SAs), the viral receptors on host cells, and for fusion of the viral and host cell membranes on endocytosis. NA is a sialidase responsible for cleaving SAs from host cells and virus particles [5, 6]. HA is cleaved into HA1 and HA2; HA1 is the major target of human immunity against the influenza A virus [7, 8]. Moreover, when newly formed virus particles bud from the host cell membrane after virus replication, NA present on the virus membrane facilitates the release of particles. A balance of competent HA and NA activities is therefore critical [9].Conversely, influenza viruses escape specific immunity induced by past infections by continuously changing the antigenicity through point mutations (drift) [10]. Many of the positively selected sites are positioned at or close to the antigenic epitopes of the HA protein, suggesting a direct role in immunological selection. Evolutionarily fit strains are selected by replicase misincorporation, which occur randomly throughout the genome. Therefore, highly variant domains are probable signs of immunological selection [11]. When a pandemic emerges through antigenic shift, it can spread globally because there is no protective antibody against the new viral strain in most of the human population [12].

Hence, the aim of this study was to elucidate the genetic variability and molecular evolution of influenza A(H1N1)pdm09 strains based on sequence analysis of the full HA1 and NA gene segments during two consecutive seasons between 2009 and 2011 in Sendai, Japan.

## Materials and methods

### Specimen collection and virus isolation

Nasopharyngeal swabs were collected from patients with influenza-like illnesses (with written informed consent), who visited outpatient clinics in Sendai City, Japan, between September 2009 and April 2011. Samples collected from 36th in 2009 to 16th in 2010 Epi-week were defined as season 2009–2010, and from 50th in 2010 to 5th in 2011 Epi-week as 2010–2011 season (here Epi-week was followed according to Japan’s Infectious Diseases Surveillance Center (IDSC) [13]. Clinical specimens were inoculated into Madin–Darby canine kidney cells with 3.5 μL/mL trypsin. The samples with a positive cytopathic effect (CPE) were confirmed to be infected with A(H1N1)pdm09 by the hemagglutination inhibition (HI) test and polymerase chain reaction (PCR). In the 2009–2010 season, which was described as the peak phase (October 2009 to January 2010) in Japan [14], 227 isolates for positive A(H1N1)pdm09 were detected, and among them, 43 representative isolates were randomly selected. In the 2010–2011 season, all 32 isolates positive for A(H1N1)pdm09 were included. Thus, a total of 75 isolates were finally selected to analyze the genetic changes in the HA1 (nucleotides 52–1,029) and NA (nucleotides 1–1,395) genes.

### Genomic sequence analysis

Viral RNA was extracted from the culture supernatants using a QIAamp viral RNA mini kit (Qiagen, Valencia, CA, USA).Viral RNA was reverse-transcribed into complementary DNA(cDNA) using the influenza A generic primer Uni12, as described previously [15]. PCR was performed using obtained cDNA and a primer set to amplify all segments, as reported previously [16] for the 2009, 2010, and 2011 viruses. The HA1 and NA gene segments were amplified using the National Institute of Technology and Evaluation and National Institute of Infectious Diseases of Japan primers (HA primerHA-1 9F ATACGACTAGCAAAAGCAGGGG and 13R TGCTCATTTTGATGGTGATAACCG; NA primer 16F AGCAAAAGCAGGAGT and 16R AGTAGAAACAAGGAG) [17], resulting in 1075- and 1446-bp amplicons, respectively. After purification of the PCR product using either a SUPREC-PCR kit (TaKaRa Bio Inc., Shiga, Japan) or a QIAquick PCR purification kit (QIAGEN), templates were labeled by carrying out a cycle-sequencing reaction using BigDye Terminator ver. 3.1 (Applied Biosystems, Foster City, CA, USA), and the products were analyzed using an automatic sequencer (3730xl Genetic Analyzer, Applied Biosystems) according to the manufacturer’s instructions. Full-length HA1 and full NA gene sequences were used for further analysis.

### Evolutionary rate

The evolutionary rate was analyzed for Sendai isolates’ sequences with a linear regression model comparing the number of substitutions/site/year with A/California/07/09 A(H1N1)pdm09strain. Periods of evolutionary rates were calculated using time interval in Epi-weeks which were described previously. All results were based on pairwise analysis, which was performed using the Maximum Composite Likelihood method in Molecular Evolutionary Genetics Analysis (MEGA) version 5 as described previously [18]. The significance of correlations was estimated using Pearson correlation.

### Phylogenetic tree analysis

In addition to the sequence data analyzed for the 2009–2011 A(H1N1)pdm09 viruses of Sendai isolates [DDBJ: AB779341–AB779490], all reference data were obtained from the influenza sequence database (Influenza Virus Resource: http://www.ncbi.nlm.nih.gov/genomes/FLU/FLU.html and The Global Initiative on Sharing All Influenza Data database)(Supplementary Table 1) [19, 20]. The references strains that we included were obtained mainly from the other prefectures of Japan and also its neighboring countries like China, Taiwan, and Korea. Nucleotide sequences were aligned using ClustalW. A phylogenetic tree was constructed by the neighbor-joining (NJ) method using MEGA ver. 5 [21]. We analyzed the phylogenetic relationships of the nucleotide sequences of HA1 and NA (complete open-reading frames) from 75 virus isolates collected in Sendai and compared the data with those of global isolates collected between April 2009 and April 2011 by the NJ method, with bootstrap values calculated from 1,000 replicates.

### Bayesian MCMC analysis

To gain insight into the evolutionary rate and mode of evolution of A(H1N1)pdm09 strains isolated in Sendai, we used a Bayesian MCMC approach as implemented in the Bayesian evolutionary analysis by sampling trees (BEAST) package v.1.7.1 [20]. Using the BEAGLE 1.0 library with the Hasegawa-Kishino-Yano (HKY) plus the general-time-reversal (GTR) plus for among-site rate heterogeneity using invariable sites (I) model, and 100 million MCMC steps, analysis of population dynamics was performed using the nonparametric Bayesian skyline plot (BSP) model, which depicts changes in genetic diversity overtime and which can be considered as a measure of effective population size assuming neutral evolution. In this study, three molecular clock models, the strict clock, the uncorrelated lognormal relaxed clock, and uncorrelated exponential relaxed clock, were used to compare the data [23–26]. The strength of model selection was assessed using a Bayes factor (BF) test, as described previously [27]. The results of the BF test revealed that both relaxed clocks fitted data were significantly better than the strict clock data. However, the uncorrelated exponential relaxed clock model was the most appropriate. We used the TRACER v1.4 program [22] from the BEAST package to visualize the result. Convergence was assessed with effective sample size values after removing 10 % of the iterations as a “burn-in.” Maximum clade credibility trees were generated using Tree Annotator from the BEAST package, and FigTree v1.3.1 (available at: http://tree.bio.ed.ac.uk) was used for visualization of the annotated trees.

### Estimation of selection pressure

Global estimates (ω) of the relative rates of nonsynonymous (dN) and synonymous (dS) substitutions averaged over the entire alignment were compared to calculate the overall strength of selection [28]. To identify the existence of positive selection pressure at the whole-gene level as well as the individual codon sites, three likelihood methods were used: single likelihood ancestor counting (SLAC), fixed-effects likelihood (FEL), and random effects likelihood (REL) approaches. According to at least one of the assay methods used (SLAC, FEL, and REL), at the specified significance levels (*P* = 0.1 and BF = 50), per gene per site dN/dS was analyzed. All analyses were performed using the online Datamonkey facility [28–30] in conjunction with the HKY 85 model for the HA gene and the Tamura–Nei model for the NA gene, with the phylogenetic tree inferred using the NJ method.

## Results

### Evolutionary rate and homology

**Fig. 1:**
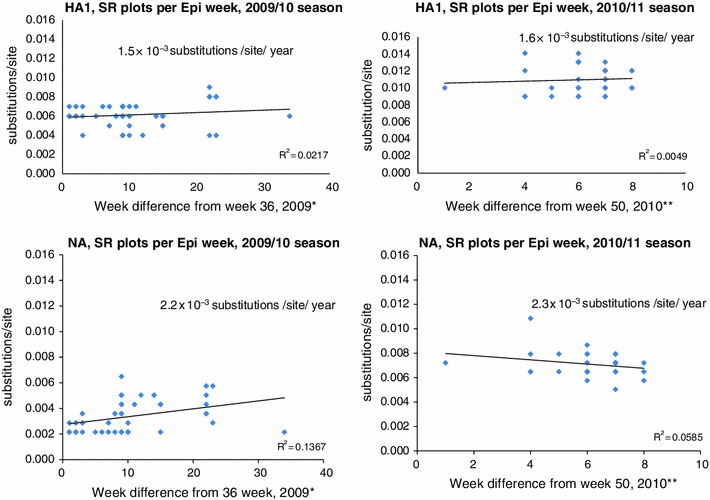
Evolutionary rate: number of nucleotide substitutions compared with the A/California/07/09 A(H1N1)pdm09strain were plotted. Evolutionary rates were calculated from the slope of the tangent of a simple regression line (number of substitutions/site) for the HA1 and NA genes for the Sendai isolates from 2009 to 2011. The square of the correlation coefficient (*r* ^2^) was estimated using Pearson’s correlation.SR, mean substitution rate. *Using time interval in week (Time starting in Epi-week from 36th in 2009 to 16th in 2010) and ** Using time interval in week (time starting in Epi-week from 50th in 2010 to 5th in 2011). Here Epi-week was followed according to Japan’s Infectious Diseases Surveillance Center

A total of 75 isolates were selected for genetic analysis of the HA1 and NA genes. The complete HA1 and NA nucleotide regions (HA1: 52–1,029 nucleotides; NA: 1–1,395 nucleotides) of the A(H1N1)pdm09 strains were amplified and sequenced. Using the standard strain A/California/07/2009(H1N1) as a reference for alignment and homology analysis, the 75 available sequenced strains exhibited high conservation, with 99.06 and 99.33 % nucleotide homologies observed for HA1 and NA, respectively. The substitution rates of nucleotides for HA1 in the 2009–2010 and 2010–2011 seasons were 1.5 × 10^−3^ and 1.6 × 10^−3^ substitutions per site per year, respectively (Fig. 1). Slopes were derived with simple linear regression, and both of them did not have enough correlation coefficient.

### Phylogenic tree analysis

**Fig. 2:**
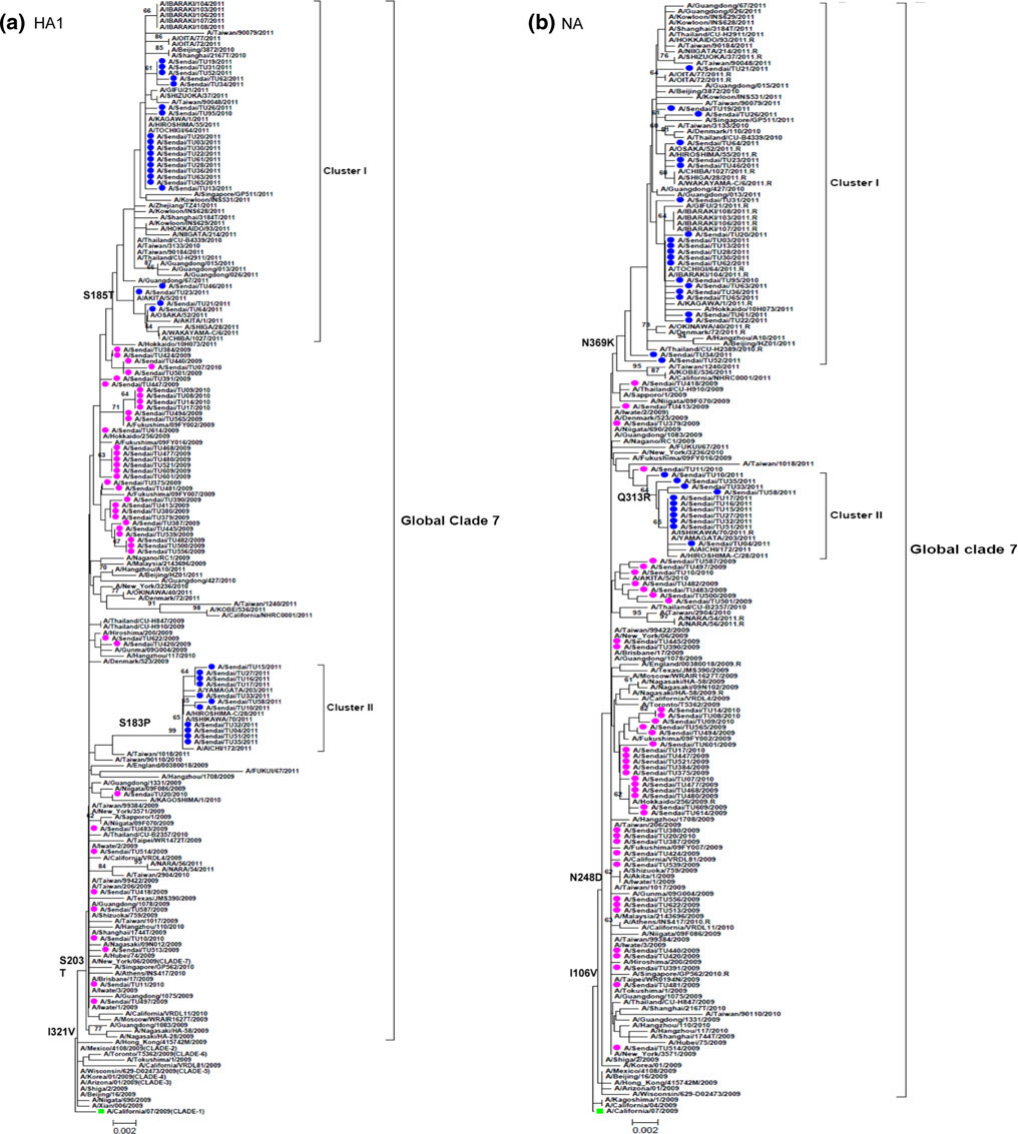
Phylogenetic trees for the **a** HA1 and **b** NA segments. The phylogenetic trees were inferred using the NJ method and bootstrap (1,000 replicates) values >60 % are shown. The *bars* at the bottom are the scales of branch lengths which show the evolutionary distances. Strains used in this study are written in *round bullet-type pink* (2009–2010 season) and *blue* (2010–2011 season). *Square green bullet* denotes vaccine strain. *Black brackets* indicate the two clusters found in 2010–2011 and global clade 7

Analysis of the phylogenetic relationship was based on the nucleotide sequences of HA1 and NA of the isolated viruses and was performed using the NJ method including other sequences from Japan and clade-specific global strains. Phylogenetic analysis of the HA1 subunit of the HA gene and the NA gene demonstrated that all the Sendai isolates clustered with HA of clade 7[31], irrespective of their year of isolation (Fig. 2a, b) [31]. The HA1 and NA genes of all the Sendai isolates for 2009–2010 did not form a monophyletic group, whereas the 2010–2011 isolates formed two distinct clusters according to the HAI phylogeny: cluster I and cluster II. These clusters could be clearly distinguished by the nonsynonymous mutations in both the HA1 and NA genes. Cluster I was characterized by the presence of the S185T mutation in the HA1 gene and the N369K mutation in the NA gene (Fig. 2a, b), whereas cluster II primarily harbored S183P mutation in the HA1gene and Q313R mutation in the NA gene. To identify a possible temporal discrete cluster in A (H1N1)pdm09 strains circulating in Sendai, we performed maximum clade credibility tree analysis (i.e., the tree sampled from the Bayesian MCMC with the highest product of individual clade probabilities). Our phylogenetic tree was consistent with our Bayesian inference data. The HA1 and NA genes of the strains isolated in Sendai during the 2010–2011 season were divergent from strains found in the 2009–2010 season (Supplementary Fig. S1 a, b). Moreover, it was evident from the Bayesian MCMC phylogenetic tree that many clusters found in the 2009–2010 season were not maintained in the 2010–2011 season. For the HA1 gene, two clusters in the 2010–2011 season were diverged from two groups of isolates in the 2009–2010 season, whereas the NA gene diverged from the same group. Moreover, the values of posterior probabilities were also high.

### Genetic diversity of the HA1 and NA genes in Sendai

**Fig. 3:**
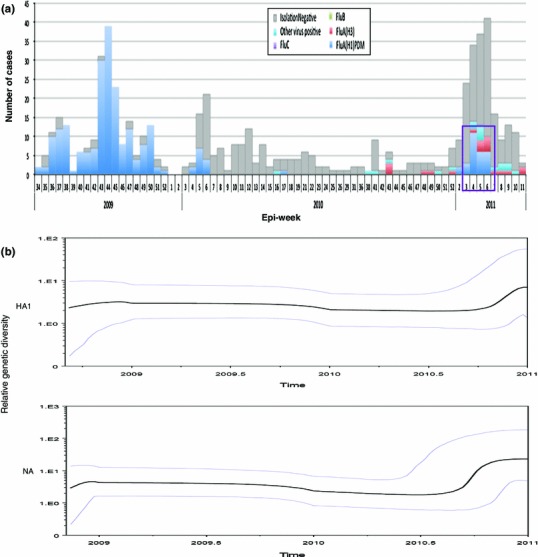
Evolutionary dynamics of the HA1 and NA genes from A(H1N1)pdm09 strains isolated in Sendai. **a** The number of A(H1N1)pdm09 cases in Epi-week since August 2009 (*blue bar*) and *purple box* indicated peak at around 4–5th Epi-weeks in 2011 from the database of Viral Respiratory Infection Surveillance conducted by Department of Virology, Tohoku University, Sendai city. **b** Changes in the genetic diversities of the HA1 and NA genes during 2009–2011 from Sendai. The *x*-axis is the year, and the *y*-axis is the relative genetic diversity. The *thick solid black line* is the median estimate, and the *pale* *blue lines* show the *upper* and *lower* bounds of the 95 % HDP interval

BSP models were used to estimate the change in the epidemic history and evolutionary dynamics of influenza A(H1N1)pdm viruses over time [24, 32]; uncertainty in the estimated parameters was evaluated using 95 % highest probability density intervals. We then used BSPs to visualize the temporal changes in genetic diversity of the HA1 and NA genes isolated in Sendai during 2009–2011 (Fig. 3). Taken together, the BSPs revealed that the Sendai influenza A(H1N1)pdm09 strains remained relatively constant in 2009–2010 season. However, a slight increase in genetic diversity was observed in the latter part of the 2010–2011 season (Fig. 3b).

### Mutation in the HA1 and NA genes

All the 75 isolates exhibited two amino acid substitutions (N1 numbering) P83S and S203T located in antigenic sites in the T cell antigen region Ca. In addition, the frequently observed amino acid substitutions(found in more than 10 isolates; Supplementary Table 2a)of the HA1 gene of the Sendai isolates were A134T, A141S, S143G, S183P, S185T, A197T, I295V, S203T, and I321V. Among these amino acid substitutions, S203T was also predominant among isolates from the United Kingdom, Japan, and other countries [31, 33]. However, amino acid substitutions in HA1at all four antigenic sites, excluding S203T, were only present in the 2010–2011 season isolates. The mutations found at the antigenic sites of the T-cell antigen region Ca and B-cell antigen region Sb were A141S, S143G, and S185T. The mutations present in RBDs and glycosylation sites were N228D, A134T, S183P, S185T, and L191I; and N228D, K119N, and Y230H (Table 1).

**Table 1:** Comparison of the number of amino acid changes in the HA1 with NA genes of the Sendai A(H1N1)pdm09 viruses during 2009–2011

Season	Hemagglutinin (HAl)		Neuraminidase (NA)	
	Number of isolates	Mutation^a^ position^b^ (total)	Number of isolates	Mutation^a^ position^c^ (total)
Sep2009–Apr2010	43	V19l(1)	43	A75V(3)^S^
		K22R(5)		A133L(1)
		V108L(1)		L140l(2)
		K171R(5)		V166F(1)
		A197T(25)		M242l(l)
		**N228D**(1)^GR^		C292S(4)
		**D274N**(2)		G382E(4)^B^
		**T310K**(4)		D416N(17)
		I333F(1)		
Sep2010–Apr2011	32	K119N(1)^G^	32	N44S(21)*^S^
		A134T(11)^**R^		I46T(10)**^S^
		A141S(11)^**T^		N50H(1)^S^
		S143G(17)^*T^		N68S(1)^S^
		S183P(11)^**R^		**R152S**(1)
		S185T(21)^*BR^		E228D(1)
		L191l(1)^R^		F231L(1)
		A197T(21)		V241l(21)*^T^
		E213D(2)		R257K(1)
		**Y230H**(4)^**G^		Q313R(11)^**^
		V234l(1)		**I321N**(1)
		N260D/H(2)		**N325K**(1)
		I295V(11)^**^		N369K(20)^*B^
				P377R(1)
				V394l(11)**
				D392S(1)^B^
				L415M(3)
				F445L(1)
Both	75	P83S(75)	75	1106V
		S203T(75)^T^		N248D
		I321V(75)		

Mutations found only in Sendai strains are written in bold

* Signature mutations of cluster I of the 2010–2011 season, ** Signature mutations of cluster II of the 2010–2011 season

^a^Ammo acid changes are described using the sequence of A/California/07/09 as a reference

^b^In these columns, “B” indicates that the site is locatedin the B-cell antigenic region. “T” indicates that the site is located in the T-cell antigenic region. “R” denotes a receptor-binding site. “G” denotes a potential glycosylation site. H1 start numbering from the amino acids DTLC, counted as 1, used elsewhere in this study

^c^In these columns, “S” indicates that the site is located in the stalk region, and the rest of the sites are located in the head region of the NA gene. B indicates that the site is located in the B-cell antigenic region. T indicates that the site is located in the T-cell antigenic region

The same analysis was performed for the NA gene of the Sendai isolates, and the results are summarized in Table 1. All the 75 isolates displayed the amino acid substitutions I106V and N248D, in line with the previously reported findings [34, 35]. Amino acid alignment of NA of the Sendai isolates against global isolates was also performed, and the results are summarized in Table 1. The frequently observed amino acid substitutions (found in more than 10 isolates; Supplementary Table 2b) in NA of the Sendai isolates included N44S, I46T, V241I, N248D, Q313R, N369K, and V394I. All mutations excluding N44S and I46T (located in the stalk region) were positioned in the globular head region of the NA gene. Moreover, for NA, the primary mutations for cluster I were N44S, V241I, and N369K, and those for cluster II were I46T, Q313R, and V394I (Table 1). Interestingly, cluster I of the 2010–2011 isolates is closely related to oseltamivir-resistant strains reported in other regions of Japan and globally (Fig. 2b). None of the isolates was positive for the known oseltamivir resistance mutation H275Y. Other amino acid substitutions, such as S247N, I223V, and I223R [36], which are also known to reduce the susceptibility to NAI, were not found in the Sendai isolates. The zanamivir resistance mutation R152K [37] was also not detected in the NA gene analysis.

### Selection analysis

Global ω values for the HA1 and NA genes were less than 1 (0.314737 and 0.257702, respectively), indicating that there is no detectable positive selection on the genes as a whole. Further site-by-site tests of positive selection helped us identify the specific sites that were not detected by the global positive selection analysis. The results obtained using the single SLAC and FEL methods were similar for the HA1 gene but not for the NA gene (Table 2). One positively selected site in the HA gene was identified at amino acid position 197, which is a receptor-binding site identified by the REL method. FEL analysis of the NA gene sequences revealed that position 46 was under positive selection (Table 2).

**Table 2:** Positively selected sites among the Sendai isolates between 2009 and 2011

Gene	Domain	Position	dN/dS^a^	Normalized [dN–dS]^b^	Posterior probability	Bayes factor	SLAC	FEL	REL
HA1	Receptor-binding domains	197		3.90714	0.991239	70.5954	N/D	N/D	
NA	Stalk	46	Infinite				N/D	0.0839931^c^	N/D

*N/D* not detected

^a^dN/dS was calculated using the FEL method

^b^Normalized [dN−dS] was calculated using the REL method

^c^The significance of the FEL result for positive selection levels is given as the *P* value

## Discussion

Analyzing the evolution of A(H1N1)pdm09 strains is important for understanding the evolutionary process of the pandemic virus, which could be different from those of seasonal influenza strains. This analysis will help us understand the emergence and spread of antigenic variants and antiviral-resistant strains of A(H1N1)pdm09 circulating in this region. Moreover, it will also help us clarify the genetic and antigenic relationships between local isolates and vaccine strains. In this study, the HA substitution rate was in line with our previous findings where the substitution rates for seasonal H1N1 and A(H1N1)pdm09 were 1.9 × 10^−3^, and 0.9 × 10^−3^ substitutions/site/year, respectively [18]. We observed only for two seasons which may not be sufficient for setting up the evolutionary rate, and this was reflected by low correlation coefficient. Phylogenetic analysis of the HA1 gene revealed that all Sendai strains were classified as clade 7, which remained the predominant circulating strains globally [31, 38]. Yet a wide variety of diversification was observed in Sendai isolates within clade 7. Moreover, the viruses from Sendai collected in the 2009–2010 season shown represented a heterogeneous cluster similar to that observed in other parts of the world [31, 39]. The absence of a monophyletic branch of the 2009–2010 viruses from Sendai suggests multiple introductions of H1N1pdm09 into this population. Phylogenetic analysis involving Bayesian MCMC methods using Sendai isolates also revealed that the short tree branch lengths had relatively rapid diversification during the peak phase (October 2009–January 2010) in Japan, whereas the longer branches in the 2010–2011 season did not have such rapid diversification (Supplementary Fig. S1 a, b). This result suggests that, there was a rapid divergence of early pandemic strains, and thereafter divergence rate decreases as the influenza virus gradually adapts to human host.

BSPs illustrated an initial decrease in the genetic diversity around September 2009, perhaps suggesting some impact of public health measures implemented worldwide at that time. However, the small increase in genetic diversity observed in 2011 in both genes (Fig. 3b) corresponded to the peak (at 4–5th Epi-week) of 2011, according to the database of the Viral Respiratory Infection Surveillance conducted by Department of Virology, Tohoku University, Sendai city (Fig. 3a). Of note, A134T, A141S, S183P, Y230H, and I295V were found in the isolates collected during that peak period (cluster II in Fig. 2a). Our data also supported that the lower number of amino acid changes in antigenic sites might verify the low selection pressure in the 2009–2010 season. Moreover, the higher genetic diversity in the latter part of the 2010–2011 season might have been caused by antigenic drifts under the selective pressure of herd immunity, because a large proportion of the human population had been infected with A(H1N1)pdm09 [40, 41].

Four antigenic sites (Ca, Cb, Sa, and Sb) for antibody recognition have been identified [31, 33] for the HA gene of A(H1N1)pdm09.With regard to the HA gene sequences of the Sendai isolates, minor changes were observed at the Ca and Sb positions. In the 2009–2010 isolates, no mutation was found in any of the four antigenic sites. However, all cluster I isolates from 2010 to 2011 shared the amino acid substitution S185T. This substitution displayed similar antigenicity as the vaccine strain A/California/7/2009 [42, 43]. It was previously described that adding an extra methyl group to the side chain may help in stabilizing the loop region in its surrounding environment, which may be responsible for the global success of circulating clade 7 viruses [31, 38]. Moreover, this small change in the side chain appears without a marked effect on the structure of HA [31, 38].

In addition, amino acid substitutions at T-cell antigenic sites, i.e., A141S and S143G, which were identified in the present study, have not been described in previous studies. Three highly conserved loops were identified in the analysis of the RBD [44], the 220-loop (residues 218–225). We also identified the A134T substitution in cluster I of the 2010–2011 viruses in the 130-loop (residues 131–135) (Table 1). Most importantly, the 190 helix (residues 184–191), which has the highest number of variations among predicted antigenic sites, was also observed in the Sendai isolates. S185T, S183P, and L191I were observed in the 2010–2011 season (Table 1). Furthermore, substitution of serine at position 183 reduced the receptor-binding ability of the H1N1pdm09 virus, resulting in a less virulent phenotype that remains to be clarified [45, 46].

The NA gene of all seasonal influenza H1N1 strains carried N248, but in the case of the A(H1N1)pdm09 strains, excluding some early strains, this amino acid is substituted with D. This substitution may be associated with oseltamivir resistance, as N248 is located near H275 [14, 47]. Another substitution at position V106I was also reported previously in isolates from Japan as well as other countries [14, 47]. Two primary mutations were observed in the stalk region of the NA gene in the Sendai isolates in the 2010–2011 season. Among them, I46T in cluster II was identified as a positively selected site. However, some other cluster-specific mutations in this study were present in the globular head, and none of them was present in the functional enzyme active sites that were found to be largely conserved among influenza A strains [35].

Influenza virus RNA has a high rate of spontaneous mutation due to the absence of viral RNA polymerase [48]. This high mutation rate facilitates escape from the immunity [48]. Moreover, for influenza viruses, positive selection for immune escape variants is believed to be generated at the population level with low levels of host cross-immunity among antigenic variants [49], which may affect the effectiveness of currently available vaccines. Our selection pressure analysis revealed that position 197 of the positively selected sites was located at the antigenic epitopes of the HA protein. This mutation was mainly present in the isolates, which were mostly collected after the 2010–2011 peak, suggesting a direct role of this mutation in immunological selection. A previous study also indicated that sites 138, 186, 190, 194, 225, 226, and 228 in HA1 are the key positions concerning its receptor-binding properties, and this might favor the inter-host transmission of the viruses from pigs to humans [50]. However, none of these positions was positively selected among the Sendai isolates, excluding position 197, which was not detected previously. Of note, this position is near amino acid position 190 in the 1918 virus HA, which has specific receptor-binding specificity [51, 52] and also plays a role in viral transmission [53]. NA sites at amino acid position 46 were under positive selection. A previous study also identified positions 46 as a positively selected site for viruses from North American swine [54]. These positions have a potential glycosylation site located in the T-cell antigenic regions, and it is associated with host adaptation after the virus is introduced from birds to humans [55]. Threonine was present in position 46 in all seasonal influenza virus strains, whereas for pandemic (H1N1) 2009, this amino acid was shifted to isoleucine. Interestingly, isolates in cluster II of the 2010–2011 season carried threonine at this position, whereas cluster I was closely related to oseltamivir-resistant strains of other countries.

## Conclusion

Our observation emphasizes the importance of continuous monitoring of A(H1N1)pdm09 strains for upcoming seasons. It is crucial to carefully monitor the underlying evolutionary changes in the virus in different geographical areas to get better selection of the vaccine strain of A(H1N1)pdm09 strains.

## Electronic supplementary material

Below is the link to the electronic supplementary material.

Supplementary material 1 (PPTX 240 kb)

Supplementary material 2 (DOCX 22 kb)

Supplementary material 3 (PPTX 77 kb)
