# Supplementary material for: Molecular evolution of the hemagglutinin and neuraminidase genes of pandemic (H1N1) 2009 influenza viruses in Sendai, Japan, during 2009–2011
Source: Virus Genes. 2013 Sep 29;47(3):456–66. doi: 10.1007/s11262-013-0980-5 (PMC3834170; doi:10.1007/s11262-013-0980-5)
Supplement: Supplementary file 1 — Supplementary material 1 (PPTX 240 kb) [file 11262_2013_980_MOESM1_ESM.pptx]

## Slide 1
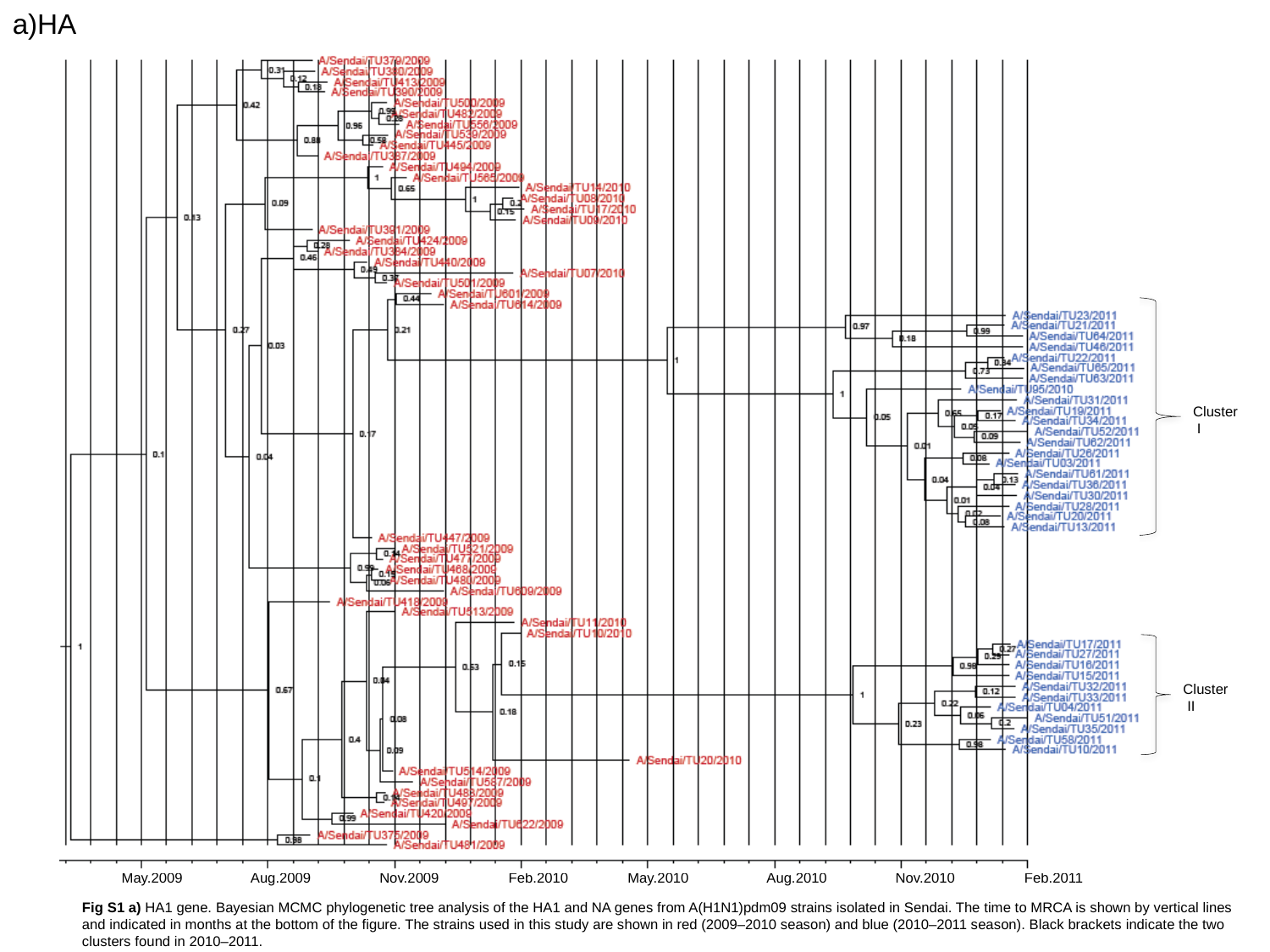

a)HA
Cluster
 I
Cluster
 II
May.2009
Aug.2009
Nov.2009
Feb.2010
May.2010
Aug.2010
Nov.2010
Feb.2011
Fig S1 a) HA1 gene. Bayesian MCMC phylogenetic tree analysis of the HA1 and NA genes from A(H1N1)pdm09 strains isolated in Sendai. The time to MRCA is shown by vertical lines and indicated in months at the bottom of the figure. The strains used in this study are shown in red (2009–2010 season) and blue (2010–2011 season). Black brackets indicate the two clusters found in 2010–2011.

## Slide 2
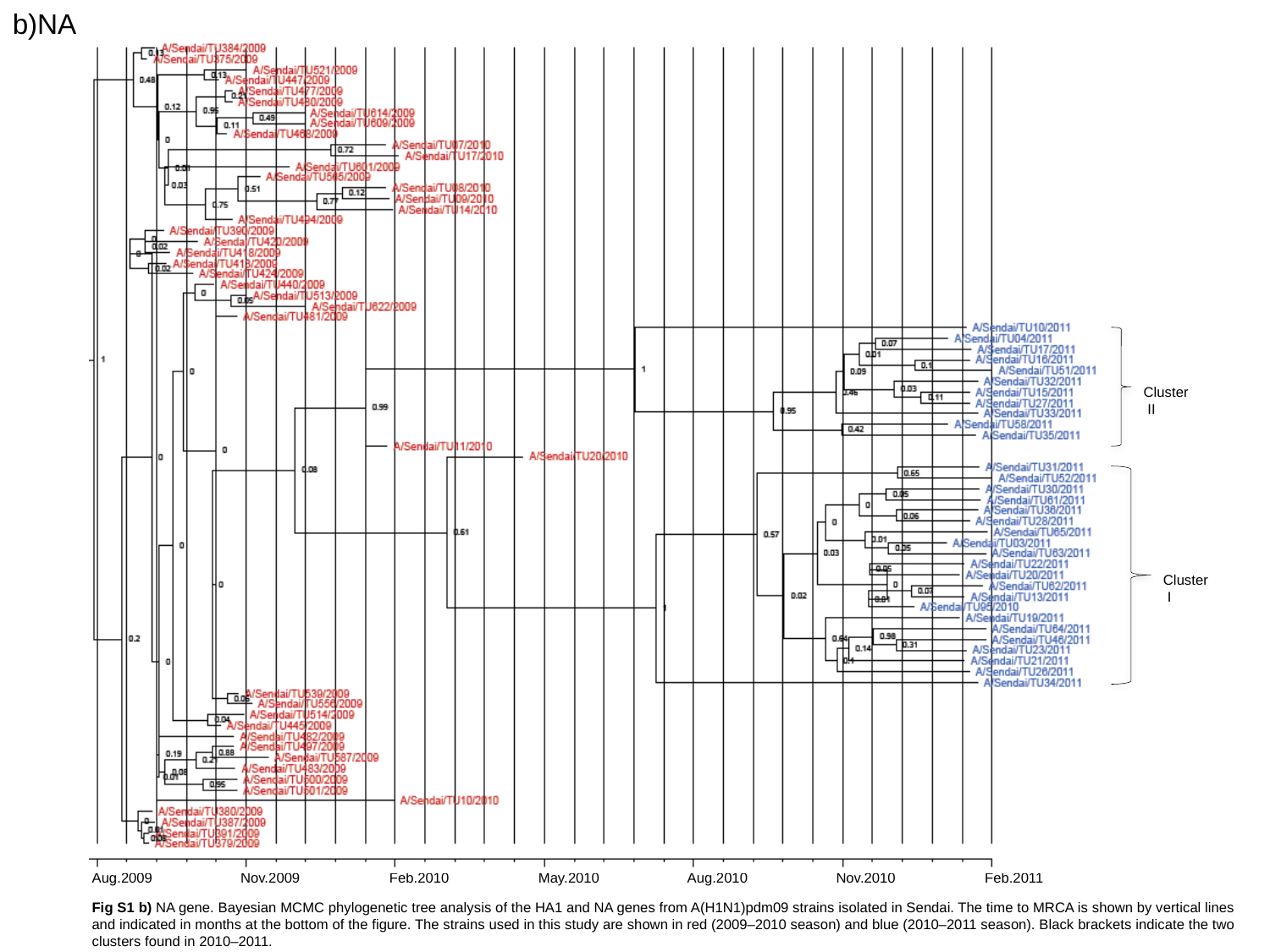

b)NA
Cluster
 II
Cluster
 I
Aug.2009
Nov.2009
Feb.2010
May.2010
Aug.2010
Nov.2010
Feb.2011
Fig S1 b) NA gene. Bayesian MCMC phylogenetic tree analysis of the HA1 and NA genes from A(H1N1)pdm09 strains isolated in Sendai. The time to MRCA is shown by vertical lines and indicated in months at the bottom of the figure. The strains used in this study are shown in red (2009–2010 season) and blue (2010–2011 season). Black brackets indicate the two clusters found in 2010–2011.
