# Supplementary material for: Molecular evolution of the hemagglutinin and neuraminidase genes of pandemic (H1N1) 2009 influenza viruses in Sendai, Japan, during 2009–2011
Source: Virus Genes. 2013 Sep 29;47(3):456–66. doi: 10.1007/s11262-013-0980-5 (PMC3834170; doi:10.1007/s11262-013-0980-5)
Supplement: Supplementary file 2 — Supplementary material 2 (DOCX 22 kb) [file 11262_2013_980_MOESM2_ESM.docx]

**Supplementary Table 1: List of accession numbers**

The file contains a list of accession numbers of the sequencing data analyzed.

| **Study isolates accession number:** AB779341-AB779490 | | |
| --- | --- | --- |
|  |  |  |
| **References strain’s accession number** | | |
| **Strain** | **Accession number** |  |
|  | **HA1** | **NA** |
| A/Hokkaido/10H073/2011 | JN790355 | JN790401 |
| A/New_York/3571/2009 | CY044869 | CY044871 |
| A/Mexico/4108/2009 | GQ149689 | GQ149688 |
| A/Korea/01/2009 | GQ131023 | GQ132185 |
| A/Arizona/01/2009 | GQ117067 | GQ117064 |
| A/California/07/2009 | KC781785 | KC781784 |
| A/Wisconsin/629-D02473/2009 | CY046283 | CY046285 |
| A/New_York/06/2009 | GQ168851 | FJ984340 |
| A/FUKUI/67/2011 | GISAID HA EPI321458 | GISAID NA EPI321457 |
| A/HOKKAIDO/93/2011 | GISAID HA EPI321482 | GISAID NA EPI3214817 |
| A/TOCHIGI/64/2011 | . GISAID HA EPI321476 | GISAID NA EPI321475 |
| A/IBARAKI/108/2011 | GISAID HA EPI321472 | GISAID NA EPI321471 |
| A/IBARAKI/107/2011 | GISAID HA EPI321470 | GISAID NA EPI321469 |
| A/IBARAKI/106/2011 | GISAID HA EPI321468 | GISAID NA EPI321467 |
| A/IBARAKI/104/2011 | GISAID HA EPI321466 | GISAID NA EPI321465 |
| A/IBARAKI/103/2011 | GISAID HA EPI321464 | GISAID NA EPI321463 |
| A/OITA/77/2011 | GISAID HA EPI321434 | GISAID NA EPI321433 |
| A/OITA/72/2011 | GISAID HA EPI321430 | GISAID NA EPI321429 |
| A/AKITA/5/2011 | GISAID HA EPI321418 | GISAID NA EPI321417 |
| A/GIFU/21/2011 | GISAID HA EPI321474 | GISAID NA EPI321473 |
| A/WAKAYAMA-C/6/2011 | GISAID HA EPI321425 | GISAID NA EPI321424 |
| A/CHIBA/1027/2011 | GISAID HA EPI321438 | GISAID NA EPI321437 |
| A/SHIGA/28/2011 | GISAID HA EPI321449 | GISAID NA EPI321448 |
| A/HIROSHIMA/55/2011 | GISAID HA EPI320266 | GISAID NA EPI320265 |
| A/NIIGATA/214/2011 | GISAID HA EPI320247 | GISAID NA EPI320246 |
| A/OSAKA/52/2011 | GISAID HA EPI320269 | GISAID NA EPI320268 |
| A/OKINAWA/40/2011 | GISAID HA EPI320274 | GISAID NA EPI320273 |
| A/SHIZUOKA/37/2011 | GISAID HA EPI320245 | GISAID NA EPI320244 |
| A/ISHIKAWA/70/2011 | GISAID HA EPI321456 | GISAID NA EPI321455 |
| A/NARA/56/2011 | GISAID HA EPI321488 | GISAID NA EPI321487 |
| A/KOBE/536/2011 | GISAID HA EPI320259 | GISAID NA EPI320258 |
| A/KAGOSHIMA/1/2010 | GQ365436 | GQ365438 |
| A/AKITA/1/2011 | GQ365410 | GQ365412 |
| A/AICHI/172/2011 | GISAID HA EPI321480 | GISAID NA EPI321479 |
| A/YAMAGATA/203/2011 | GISAID HA EPI321427 |  |
| A/HIROSHIMA-C/28/2011 | GISAID HA EPI320243 | GISAID NA EPI320242 |
| A/Athens/INS417/2010 | CY071295 | CY071297 |
| A/California/VRDL11/2010 | CY063187 | CY063189 |
| A/New York/3236/2010 | CY062170 | CY062172 |
| A/Singapore/GP562/2010 | CY063830 | CY063832 |
| A/California/NHRC0001/2011 | CY092880 | CY092882 |
| A/Thailand/CU-H2911/2011 | CY089463 | CY089465 |
| A/NARA/54/2011 | GISAID HA EPI321443 | GISAID NA EPI321442 |
| A/KAGAWA/1/2011 | GISAID HA EPI321441 | GISAID NA EPI321440 |
| A/Singapore/GP511/2011 | CY091692 | CY091693 |
| A/Denmark/72/2011 | CY090808 | CY090810 |
| A/Denmark/523/2009 | CY043334 | CY043336 |
| A/Sapporo/1/2009 | GQ365446 | GQ365448 |
| A/Nagano/RC1/2009 | AB538389 | AB538391 |
| A/Shizuoka/759/2009 | GQ334346 | GQ334348 |
| A/Tokushima/1/2009 | GQ287625 | GQ287626 |
| A/Brisbane/17/2009 | GQ160610 | GQ160611 |
| A/Niigata/690/2009 | GU014798. | GU014799 |
| A/England/00380018/2009 | CY057040 | CY067720 |
| A/Texas/JMS390/2009 | CY061035 | CY061037 |
| A/Nagasaki/HA-58/2009 | AB536769 | AB537490 |
| A/Shiga/2/2009 | GQ287621 | GQ287622 |
| A/Iwate/3/2009 | GU014790 | GU014791 |
| A/Moscow/WRAIR1627T/2009 | CY083248 | CY083250 |
| A/California/VRDL81/2009 | CY062210 | CY062212 |
| A/California/VRDL4/2009 | CY054707 | CY054709 |
| A/Hokkaido/256/2009 | AB576885 | AB576884 |
| A/Denmark/110/2010 |  | HQ880597 |
| A/Thailand/CU-H2389/2010 | CY088823 | CY082967 |
| A/Kagoshima/1/2009 | GQ365436 | GQ365438 |
| A/California/04/2009 | GQ117044 | FJ966084 |
| A/Beijing/16/2009 | HQ698627 | HQ698629 |
| A/Nagasaki/HA-28/2009 | AB530475 | AB537490 |
| A/Guangdong/1331/2009 | CY058820 | CY058821 |
| A/Guangdong/1075/2009 | CY064428 | CY064429 |
| A/Guangdong/1078/2009 | CY064430 | CY064431 |
| A/Guangdong/1083/2009 | CY064432 | CY064433 |
| A/Niigata/09F070/2009 | CY066023 | CY066024 |
| A/Fukushima/09FY002/2009 | CY066029 | CY066026 |
| A/Fukushima/09FY007/2009 | CY066033 | CY066030 |
| A/Fukushima/09FY016/2009 | CY066037 | CY066034 |
| A/Gunma/09G004/2009 | CY066059 | CY066038 |
| A/Nagasaki/09N012/2009 | CY066135 | CY066060 |
| A/Taipei/WR1472T/2009 | CY071626 | CY066170 |
| A/Thailand/CU-H847/2009 | CY075006 | CY071412 |
| A/Thailand/CU-H910/2009 | CY075014 | CY075008 |
| A/Thailand/CU-B2357/2010 | CY080299 | CY075016 |
| A/Beijing/3872/2010 | CY081580 | CY080301 |
| A/Thailand/CU-B4339/2010 | CY089437 | CY081581 |
| A/Hubei/74/2009 | CY095862 | CY089439 |
| A/Malaysia/2143696/2009 | CY119346 | CY095872 |
| A/Guangdong/67/2011 | CY120950 | CY119348 |
| A/Kowloon/INS628/2011 | CY129573 | CY120951 |
| A/Kowloon/INS531/2011 | CY129582 | CY129575 |
| A/Kowloon/INS629/2011 | CY129990 | CY129584 |
| A/Iwate/1/2009 | GQ365426 | CY129992 |
| A/Iwate/2/2009 | GQ365428 | GQ365427 |
| A/Hiroshima/200/2009 | GU014780 | GQ365430 |
| A/HongKong/415742M/2009 | GU931802 | GU014781 |
| A/Xian/006/2009 | HM006717 | GU931806 |
| A/Beijing/HZ01/2011 | JF816658 | JF316715 |
| A/Guangdong/427/2010 | JF929758 | JF929803 |
| A/Guangdong/013/2011 | JF929768 | JF929817 |
| A/Guangdong/015/2011 | JF929769 | JF929818 |
| A/Guangdong/026/2011 | JF929770 | JF929819 |
| A/Taiwan/1017/2009 | JN187137 | JN187195 |
| A/Taiwan/1018/2011 | JN187143 | JN187201 |
| A/Taiwan/99384/2009 | JN381239 | JN381379 |
| A/Taiwan/99422/2009 | JN381240 | JN381380 |
| A/Taiwan/90110/2010 | JN381271 | JN381411 |
| A/Taiwan/2904/2010 | JN381280 | JN381420 |
| A/Taiwan/3133/2010 | JN381288 | JN381428 |
| A/Taiwan/90048/2011 | JN381307 | JN381447 |
| A/Taiwan/90079/2011 | JN381312 | JN381452 |
| A/Taiwan/90184/2011 | JN381323 | JN381463 |
| A/Shanghai/3184T/2011 | JN631049 | JN631040 |
| A/Shanghai/2167T/2010 | JN631052 | JN631041 |
| A/Shanghai/1744T/2009 | JN631054 | JN631046 |
| A/Hangzhou/1708/2009 | JQ364992 | JN006474 |
| A/Hangzhou/110/2010 | JQ365004 | JN006486 |
| A/Hangzhou/117/2010 | JQ365007 | JN006489 |
| A/Hangzhou/A10/2011 | JQ365016 | JN006499 |
| A/Taiwan/1240/2011 | JQ693689 | JQ693709 |
| A/Zhejiang/TZ41/2011 | JQ796824 |  |
| A/Taiwan/206/2009 |  | CY053508 |
