# Supplementary material for: Molecular evolution of the hemagglutinin and neuraminidase genes of pandemic (H1N1) 2009 influenza viruses in Sendai, Japan, during 2009–2011
Source: Virus Genes. 2013 Sep 29;47(3):456–66. doi: 10.1007/s11262-013-0980-5 (PMC3834170; doi:10.1007/s11262-013-0980-5)
Supplement: Supplementary file 3 — Supplementary material 3 (PPTX 77 kb) [file 11262_2013_980_MOESM3_ESM.pptx]

## Slide 1
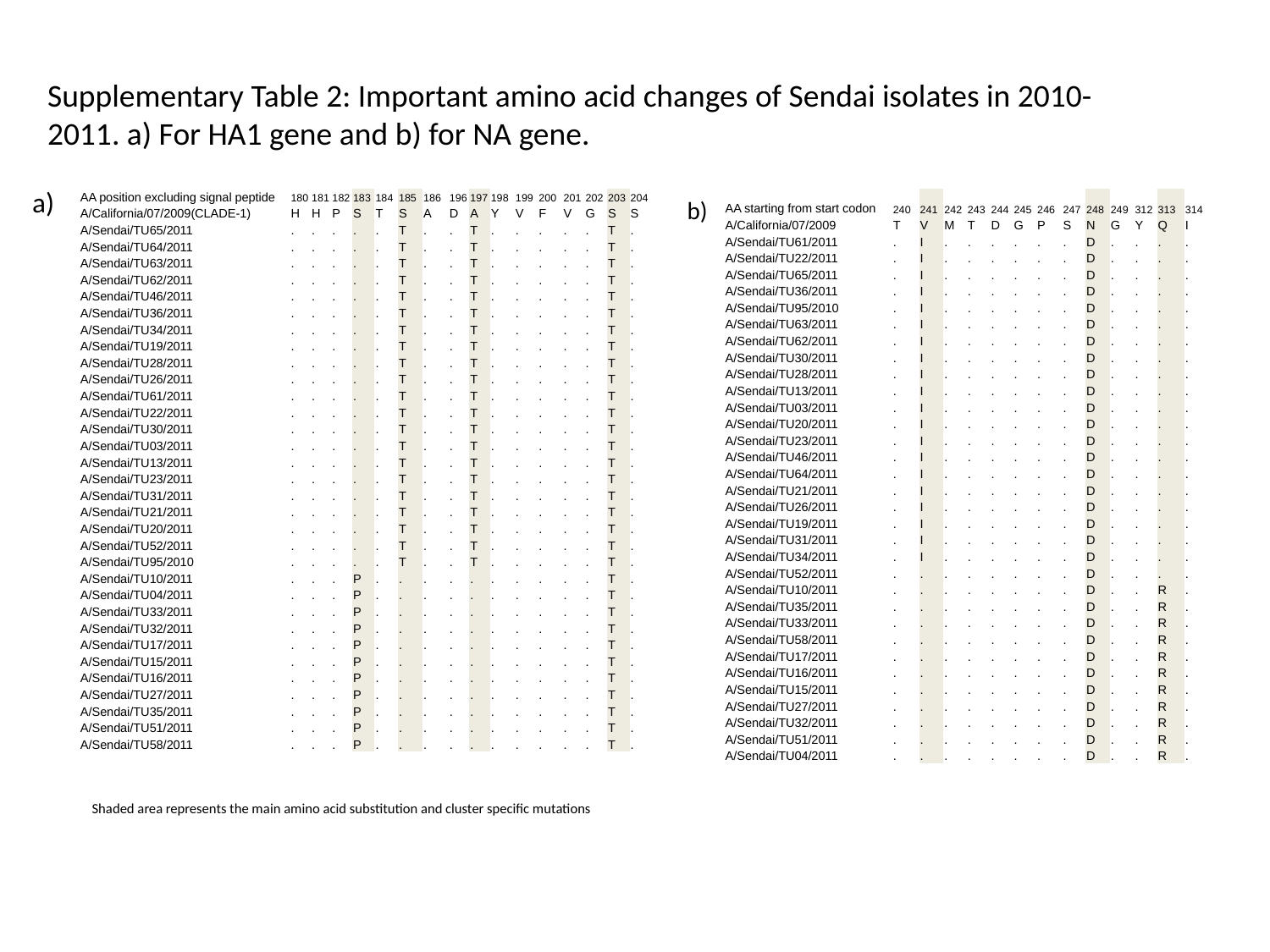

Supplementary Table 2: Important amino acid changes of Sendai isolates in 2010-2011. a) For HA1 gene and b) for NA gene.
a)
| AA position excluding signal peptide | 180 | 181 | 182 | 183 | 184 | 185 | 186 | 196 | 197 | 198 | 199 | 200 | 201 | 202 | 203 | 204 |
| --- | --- | --- | --- | --- | --- | --- | --- | --- | --- | --- | --- | --- | --- | --- | --- | --- |
| A/California/07/2009(CLADE-1) | H | H | P | S | T | S | A | D | A | Y | V | F | V | G | S | S |
| A/Sendai/TU65/2011 | . | . | . | . | . | T | . | . | T | . | . | . | . | . | T | . |
| A/Sendai/TU64/2011 | . | . | . | . | . | T | . | . | T | . | . | . | . | . | T | . |
| A/Sendai/TU63/2011 | . | . | . | . | . | T | . | . | T | . | . | . | . | . | T | . |
| A/Sendai/TU62/2011 | . | . | . | . | . | T | . | . | T | . | . | . | . | . | T | . |
| A/Sendai/TU46/2011 | . | . | . | . | . | T | . | . | T | . | . | . | . | . | T | . |
| A/Sendai/TU36/2011 | . | . | . | . | . | T | . | . | T | . | . | . | . | . | T | . |
| A/Sendai/TU34/2011 | . | . | . | . | . | T | . | . | T | . | . | . | . | . | T | . |
| A/Sendai/TU19/2011 | . | . | . | . | . | T | . | . | T | . | . | . | . | . | T | . |
| A/Sendai/TU28/2011 | . | . | . | . | . | T | . | . | T | . | . | . | . | . | T | . |
| A/Sendai/TU26/2011 | . | . | . | . | . | T | . | . | T | . | . | . | . | . | T | . |
| A/Sendai/TU61/2011 | . | . | . | . | . | T | . | . | T | . | . | . | . | . | T | . |
| A/Sendai/TU22/2011 | . | . | . | . | . | T | . | . | T | . | . | . | . | . | T | . |
| A/Sendai/TU30/2011 | . | . | . | . | . | T | . | . | T | . | . | . | . | . | T | . |
| A/Sendai/TU03/2011 | . | . | . | . | . | T | . | . | T | . | . | . | . | . | T | . |
| A/Sendai/TU13/2011 | . | . | . | . | . | T | . | . | T | . | . | . | . | . | T | . |
| A/Sendai/TU23/2011 | . | . | . | . | . | T | . | . | T | . | . | . | . | . | T | . |
| A/Sendai/TU31/2011 | . | . | . | . | . | T | . | . | T | . | . | . | . | . | T | . |
| A/Sendai/TU21/2011 | . | . | . | . | . | T | . | . | T | . | . | . | . | . | T | . |
| A/Sendai/TU20/2011 | . | . | . | . | . | T | . | . | T | . | . | . | . | . | T | . |
| A/Sendai/TU52/2011 | . | . | . | . | . | T | . | . | T | . | . | . | . | . | T | . |
| A/Sendai/TU95/2010 | . | . | . | . | . | T | . | . | T | . | . | . | . | . | T | . |
| A/Sendai/TU10/2011 | . | . | . | P | . | . | . | . | . | . | . | . | . | . | T | . |
| A/Sendai/TU04/2011 | . | . | . | P | . | . | . | . | . | . | . | . | . | . | T | . |
| A/Sendai/TU33/2011 | . | . | . | P | . | . | . | . | . | . | . | . | . | . | T | . |
| A/Sendai/TU32/2011 | . | . | . | P | . | . | . | . | . | . | . | . | . | . | T | . |
| A/Sendai/TU17/2011 | . | . | . | P | . | . | . | . | . | . | . | . | . | . | T | . |
| A/Sendai/TU15/2011 | . | . | . | P | . | . | . | . | . | . | . | . | . | . | T | . |
| A/Sendai/TU16/2011 | . | . | . | P | . | . | . | . | . | . | . | . | . | . | T | . |
| A/Sendai/TU27/2011 | . | . | . | P | . | . | . | . | . | . | . | . | . | . | T | . |
| A/Sendai/TU35/2011 | . | . | . | P | . | . | . | . | . | . | . | . | . | . | T | . |
| A/Sendai/TU51/2011 | . | . | . | P | . | . | . | . | . | . | . | . | . | . | T | . |
| A/Sendai/TU58/2011 | . | . | . | P | . | . | . | . | . | . | . | . | . | . | T | . |
b)
| AA starting from start codon | 240 | 241 | 242 | 243 | 244 | 245 | 246 | 247 | 248 | 249 | 312 | 313 | 314 |
| --- | --- | --- | --- | --- | --- | --- | --- | --- | --- | --- | --- | --- | --- |
| A/California/07/2009 | T | V | M | T | D | G | P | S | N | G | Y | Q | I |
| A/Sendai/TU61/2011 | . | I | . | . | . | . | . | . | D | . | . | . | . |
| A/Sendai/TU22/2011 | . | I | . | . | . | . | . | . | D | . | . | . | . |
| A/Sendai/TU65/2011 | . | I | . | . | . | . | . | . | D | . | . | . | . |
| A/Sendai/TU36/2011 | . | I | . | . | . | . | . | . | D | . | . | . | . |
| A/Sendai/TU95/2010 | . | I | . | . | . | . | . | . | D | . | . | . | . |
| A/Sendai/TU63/2011 | . | I | . | . | . | . | . | . | D | . | . | . | . |
| A/Sendai/TU62/2011 | . | I | . | . | . | . | . | . | D | . | . | . | . |
| A/Sendai/TU30/2011 | . | I | . | . | . | . | . | . | D | . | . | . | . |
| A/Sendai/TU28/2011 | . | I | . | . | . | . | . | . | D | . | . | . | . |
| A/Sendai/TU13/2011 | . | I | . | . | . | . | . | . | D | . | . | . | . |
| A/Sendai/TU03/2011 | . | I | . | . | . | . | . | . | D | . | . | . | . |
| A/Sendai/TU20/2011 | . | I | . | . | . | . | . | . | D | . | . | . | . |
| A/Sendai/TU23/2011 | . | I | . | . | . | . | . | . | D | . | . | . | . |
| A/Sendai/TU46/2011 | . | I | . | . | . | . | . | . | D | . | . | . | . |
| A/Sendai/TU64/2011 | . | I | . | . | . | . | . | . | D | . | . | . | . |
| A/Sendai/TU21/2011 | . | I | . | . | . | . | . | . | D | . | . | . | . |
| A/Sendai/TU26/2011 | . | I | . | . | . | . | . | . | D | . | . | . | . |
| A/Sendai/TU19/2011 | . | I | . | . | . | . | . | . | D | . | . | . | . |
| A/Sendai/TU31/2011 | . | I | . | . | . | . | . | . | D | . | . | . | . |
| A/Sendai/TU34/2011 | . | I | . | . | . | . | . | . | D | . | . | . | . |
| A/Sendai/TU52/2011 | . | . | . | . | . | . | . | . | D | . | . | . | . |
| A/Sendai/TU10/2011 | . | . | . | . | . | . | . | . | D | . | . | R | . |
| A/Sendai/TU35/2011 | . | . | . | . | . | . | . | . | D | . | . | R | . |
| A/Sendai/TU33/2011 | . | . | . | . | . | . | . | . | D | . | . | R | . |
| A/Sendai/TU58/2011 | . | . | . | . | . | . | . | . | D | . | . | R | . |
| A/Sendai/TU17/2011 | . | . | . | . | . | . | . | . | D | . | . | R | . |
| A/Sendai/TU16/2011 | . | . | . | . | . | . | . | . | D | . | . | R | . |
| A/Sendai/TU15/2011 | . | . | . | . | . | . | . | . | D | . | . | R | . |
| A/Sendai/TU27/2011 | . | . | . | . | . | . | . | . | D | . | . | R | . |
| A/Sendai/TU32/2011 | . | . | . | . | . | . | . | . | D | . | . | R | . |
| A/Sendai/TU51/2011 | . | . | . | . | . | . | . | . | D | . | . | R | . |
| A/Sendai/TU04/2011 | . | . | . | . | . | . | . | . | D | . | . | R | . |
Shaded area represents the main amino acid substitution and cluster specific mutations
